# Influence of Surface Treatments on the Pull-Off Performance of Adhesively Bonded Polylactic Acid (PLA) Specimens Manufactured by Fused Deposition Modeling (FDM)

**DOI:** 10.3390/ma18173965

**Published:** 2025-08-24

**Authors:** Gianluca Parodo, Giuseppe Moffa, Alessandro Silvestri, Luca Sorrentino, Gabriel Testa, Sandro Turchetta

**Affiliations:** 1 Department of Engineering and Sciences, Faculty of Technological and Innovation Sciences, Universitas Mercatorum, Piazza Mattei 10, 00186 Rome, Italy; 2 Department of Civil and Mechanical Engineering, University of Cassino and Southern Lazio, 03043 Cassino, Italy; giuseppe.moffa@unicas.it (G.M.); silvestr@unicas.it (A.S.); luca.sorrentino@unicas.it (L.S.); gabriel.testa@unicas.it (G.T.); sandro.turchetta@unicas.it (S.T.); 3 European Union, European University of Technology EUt+

**Keywords:** PLA, adhesive bonding, surface treatment, epoxy adhesive, pull-off test, experimental characterization

## Abstract

This study investigates the influence of different surface treatments (namely, mechanical abrasion and solvent cleaning with isopropyl alcohol and acetone) on the adhesive bonding performance of polylactic acid (PLA) substrates produced by Fused Deposition Modeling (FDM). Pull-off tests revealed that the isopropanol-cleaned specimens achieved the highest bond strength, with an average pull-off value exceeding 8.5 MPa, compared to approximately 5.6 MPa for untreated PLA. Conversely, acetone cleaning resulted in the lowest performance (about 3.5 MPa), while mechanical abrasion yielded intermediate values of about 6 MPa. FTIR analysis confirmed that no chemical reactions occurred, although acetone and abrasion induced significant physical surface changes, unlike isopropanol, which acted as an effective cleaning agent. These findings demonstrate that surface cleanliness plays a dominant role over morphological alterations in enhancing the adhesion of PLA-based 3D-printed joints.

## 1. Introduction

Structural bonding is a widely used joining technology across various industrial sectors due to its ability to evenly distribute stresses and preserve the structural integrity of the materials involved [1] while avoiding the need for heavy fasteners or welds, enabling lightweight, sealed joints and allowing the joining of dissimilar materials [2]. Compared to other joining techniques, such as welding or bolting, adhesive bonding allows the joining of dissimilar materials while minimizing stress concentrations within the joint, ensuring high mechanical performance without significantly altering the properties of the substrates [3,4].

In the case of polymeric materials, adhesion poses a significant challenge due to their chemical inertness, low surface energy, and the possible presence of release agents or impurities that can compromise bonding quality [5,6]. Among polymers of growing interest, polylactic acid (PLA) is extensively studied for its biodegradable properties and applicability in additive manufacturing [7,8,9]. However, its adhesion to structural adhesives is often limited without proper surface preparation [10].

Fused Deposition Modeling (FDM) is one of the most widely adopted additive manufacturing technologies due to its low cost, design flexibility, and compatibility with biodegradable thermoplastics [11,12,13]. PLA is particularly attractive for its biocompatibility and ease of printing, but it often exhibits poor interfacial adhesion when bonded with adhesives [14].

The adhesion between polymeric substrates and adhesives is strongly influenced by surface interactions, which depend on factors such as surface tension, topography, and the presence of functional groups capable of forming physical or chemical bonds with the adhesive matrix. Surface treatments are commonly employed to increase roughness and modify the substrate’s chemistry to enhance bonding effectiveness. Techniques such as mechanical sanding, plasma treatment, solvent cleaning, or adhesive primers can significantly alter surface properties, improving adhesive wettability and promoting mechanical or chemical anchoring to the substrate [15,16,17,18,19,20,21].

The evaluation of adhesive joint performance is traditionally carried out through mechanical tests that assess the strength of the adhesive interface. The lap-shear test is the most commonly used method in the literature due to its simplicity and ability to directly measure the adhesive’s shear strength [17]. These mechanical tests provide a basis to compare different adhesives, substrates, and processing conditions (cure schedules, joint geometry, etc.) under controlled shear loading. However, this test may not be fully representative in cases where the applied load is predominantly normal to the bonded surface, such as in coatings, adhesive bonding on low-surface-energy materials, or substrates with particular surface finishes, like those produced via Fused Deposition Modeling (FDM). In this regard, achieving effective adhesion to polymeric substrates like PLA is particularly challenging due to its chemically inert nature. Specifically, PLA’s molecular structure (comprising methyl and carbonyl side groups) exhibits limited polar functionality, resulting in a scarcity of active sites capable of establishing strong interactions with the adhesive matrix [15].

Furthermore, PLA exhibits inherent hydrophobicity, with static water contact angles typically of 75° to 85° [10], indicating low surface energy and poor wettability by conventional adhesives. In this context, the pull-off test is a more severe and discriminating method for evaluating adhesion, as the normal stress directly forces the adhesive to detach from the substrate. This condition is particularly critical for FDM-printed polymeric materials, which inherently exhibit an inhomogeneous surface characterized by discontinuities between layers and roughness that can vary significantly depending on the printing parameters. Applying a more sensitive perpendicular load reveals the adhesive bond’s true quality and the effects of surface treatments aimed at improving wettability and mechanical anchoring. Compared to the lap-shear test, the pull-off test is less influenced by the mechanical strength of the substrate and more sensitive to the adhesion quality at the adhesive–substrate interface. This makes it particularly useful for differentiating the effectiveness of surface treatments, allowing for the measurement of maximum detachment strength and the identification of failure modes (adhesive, cohesive, or mixed). These insights are essential for understanding the mechanisms governing adhesive interactions.

This study investigates how different surface treatment strategies affect the adhesion strength of bonded joints in PLA specimens fabricated via Fused Deposition Modeling (FDM), using the pull-off test as the primary evaluation method. In particular, mechanical abrasion and solvent-based cleaning methods are examined to assess their impact on surface morphology and chemical affinity. The main goal is to determine whether the observed improvements in adhesion are driven by enhanced mechanical interlocking, chemical activation, or the synergistic contribution of both mechanisms.

The present research was developed thanks to the 3D-EcoCore project (founded by the European Union under the NextGenerationEU (PNRR) program), whose focus is the creation of new advanced sandwich structures using biodegradable and biocompatible materials produced through 3D printing. The project explores opportunities to use innovative technologies and sustainable materials to address these challenges, as discussed in the following sections.

## 2. Materials and Methods

Square plates of PLA with dimensions of 50 mm × 50 mm and a nominal thickness of 2 mm were fabricated via FDM using a Prusa XL 3D printer (Prusa Research, Prague, Czech Republic). The printing parameters are summarized in Table 1, including the nozzle temperature, print speed, layer height, and infill density. All specimens were printed in a flat orientation with the bonding surface corresponding to the top layer of the printed part.

### 2.1. Surface Characterization

Following printing, the bonding surfaces were subjected to different surface treatment protocols according to the experimental plan detailed in Table 2. Four surface preparation conditions were considered:

1. Untreated (UT): Specimens used in their original condition, directly after the FDM printing process, without any additional cleaning, surface modification, or post-processing. This condition preserves the native topography and any surface residues generated during printing.
2. Alcohol Cleaning (AL-C): The bonding surface was cleaned using 99% isopropyl alcohol (IPA) applied with lint-free wipes to remove possible contaminants and surface release agents.
3. Mechanical Abrasion (MA): The surface was abraded using P240 grit sandpaper applied manually in a circular motion. To minimize variability, all specimens were treated by the same operator using a consistent motion and fixed duration. While manual sanding may introduce some limitations in terms of reproducibility, it reflects a widely used and accessible surface preparation technique in industrial practice (particularly suitable for parts with complex geometries or when automation is not feasible). Therefore, despite its manual nature, this treatment retains practical relevance and offers valuable insights into real-world bonding conditions.
4. Acetone Cleaning (AC-C): The bonding surface was cleaned using 99% acetone applied with lint-free wipes in a uniform manner.

These four surface preparation strategies were selected to isolate different potential adhesion mechanisms. Mechanical abrasion (MA) is expected to enhance mechanical interlocking by increasing surface roughness. Isopropyl alcohol (AC) serves as a mild degreasing agent to remove contaminants and improve surface energy. Acetone treatment (AC-C), being a more aggressive solvent, may induce partial surface dissolution or reflow, potentially altering both morphology and surface chemistry. The untreated condition (UT) serves as a baseline reference. This experimental design allows for a comparative assessment of the relative contributions of surface morphology and chemical activation to adhesion strength.

Surface roughness was measured using a stylus profilometer (Taylor Hobson Intra Touch, Leicester, UK), following the ISO 21920 standard, which updates the previous ISO 4287 specification. Three profiles were recorded per specimen. Surface roughness was evaluated through the average roughness (Ra) and root-mean-square roughness (Rq), which represent the arithmetic mean and the standard deviation of the profile height variations relative to the mean line, respectively, as defined by ISO 21920.

Fourier-transform infrared (FTIR) spectroscopy was performed to investigate possible chemical and structural modifications of the PLA surface after treatment. Spectra were acquired using a PerkinElmer Spectrum Two spectrometer (PerkinElmer, Shelton, CT, USA) in attenuated total reflectance (ATR) mode. For each sample, 32 scans were collected in the spectral range of 4000–400 cm^−1^, with a resolution of 4 cm^−1^. The samples were analyzed in their solid state, with no additional preparation.

### 2.2. Adhesion Tests

The bonding was performed using a two-component structural epoxy adhesive, selected for its high mechanical strength and compatibility with thermoplastic substrates. The adhesive used for bonding was the two-part epoxy 3M EC-9323 B/A, mixed at a 2:1 weight ratio as recommended by the manufacturer. The adhesive was applied to the treated PLA surface using a 100 µm notched applicator to ensure consistent adhesive layer thickness.

**Figure 1:**
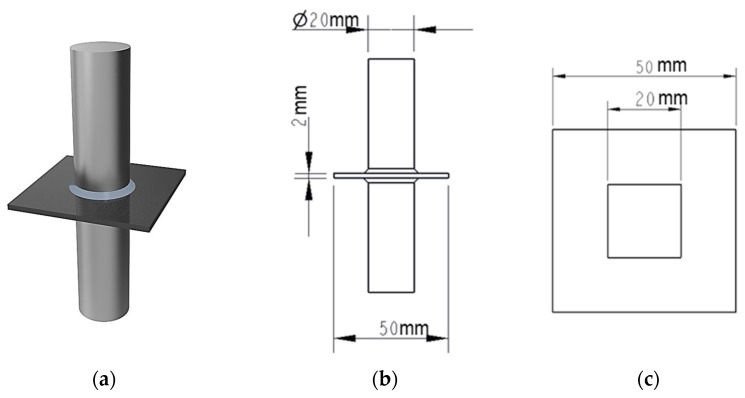
Schematic representation of the bonded assembly: (**a**) 3D view of the PLA-to-steel pull-off fixture; (**b**) 2D technical drawing with dimensions; (**c**) schematic of the confined square bonding area on one side of the PLA plate.

Bonded assemblies were fabricated by joining PLA plates to steel loading studs (cylindrical, 20 mm in diameter), which acted as pull-off fixtures, as schematically illustrated in Figure 1a,b. The bonding interface on one side involved full-surface adhesion between the PLA and the entire circular face of the steel cylinder (diameter: 20 mm). On the opposite side, the adhesive contact was deliberately confined to a square region measuring 20 mm × 20 mm (Figure 1c), achieved by masking the surrounding area of the PLA plate with tape prior to bonding. This configuration was designed to promote interfacial failure on the restricted-bonding side, where the contact area was both smaller and precisely defined. Additionally, the adhesive formed a fillet between the plate and the steel cylinders, effectively increasing the contact area. This fillet allowed for partial stress transfer through shear, enhancing the bonding strength of the junction and reducing the risk of debonding on the side where failure was not intended.

The bonded specimens were left to cure in ambient conditions for 24 h and then post-cured at 60 °C for 2 h to ensure full crosslinking. Adhesion performance was evaluated via pull-off tests using a universal testing machine equipped with a 10 kN load cell, following the guidelines of ASTM D4541. The load was applied perpendicularly to the bonded surface at a constant displacement rate of 1 mm/min until complete detachment. Five specimens were tested for each surface condition. The maximum force at detachment was recorded, and the failure mode was subsequently classified via optical analysis as adhesive, cohesive, or mixed.

## 3. Results and Discussion

### 3.1. Surface Characterization

**Figure 2:**
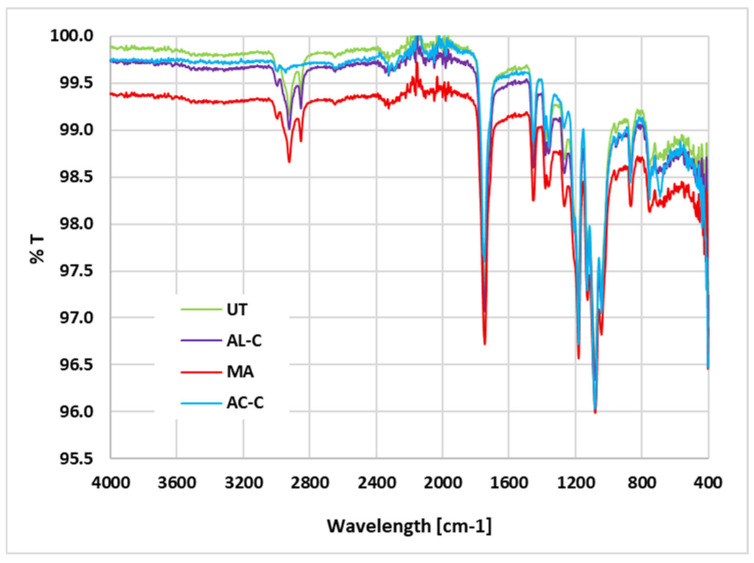
FTIR results for different surface treatments: untreated (UT); isopropyl alcohol-cleaned (AL-C); mechanical abrasion (MA); acetone-cleaned (AC-C).

The comparison of the FTIR spectra for untreated PLA (UT), acetone-treated (AC-C), isopropanol-treated (AL-C), and mechanically abraded (MA) samples is shown in Figure 2. The main spectral features are consistent with the typical infrared response of polylactic acid reported in the literature: the strong absorption band at 1750 cm^−1^ corresponds to the stretching vibration of the ester carbonyl group (C=O) [7,22], the peaks between 2945 and 2997 cm^−1^ can be associated with the asymmetric and symmetric C-H stretching of CH_3_ and CH_2_ groups [15], and the band at 1450 cm^−1^ can be assigned to CH_3_ bending, while the complex region between 1080 and 1180 cm^−1^ can be attributed to C-O-C stretching vibrations of the ester linkage [14,16].

The AC-C sample exhibits a marked increase in transmittance in the 3000–2800 cm^−1^ region, which can be ascribed to a reorientation of polymer chains at the surface, induced by acetone’s solvating action. This effect is consistent with previous studies on solvent-treated PLA surfaces, where partial chain mobility led to enhanced exposure of aliphatic groups without introducing new chemical functionalities [19]. The absence of additional peaks, particularly in the carbonyl and hydroxyl regions, confirms the lack of chemical reactions such as hydrolysis or oxidation under the applied conditions.

The AL-C spectrum is nearly identical to that of the untreated sample, in agreement with the role of isopropanol as a mild degreasing agent with negligible effect on PLA chain conformation or chemical structure [10]. Its influence is therefore limited to contaminant removal and possible minor changes in surface energy.

The MA sample shows a general decrease in transmittance, especially in the 3000–2800 cm^−1^ and 1300–1000 cm^−1^ regions, which is compatible with increased light scattering due to the roughened surface produced by abrasion. As reported by Kariž et al. [16], such morphological changes can alter spectral baseline and peak intensities without modifying chemical bonds.

Overall, the results indicate that acetone and mechanical abrasion cause detectable physical changes in the ATR-FTIR spectra, whereas isopropanol treatment leaves the surface chemistry largely unaltered. All treatments maintain the chemical identity of PLA. It should be noted that ATR-FTIR primarily probes a penetration depth in the order of 1–2 µm; thus, modifications confined to nanometric surface layers may remain undetected, as observed in other studies on solvent- and plasma-treated polymers [17].

**Figure 3:**
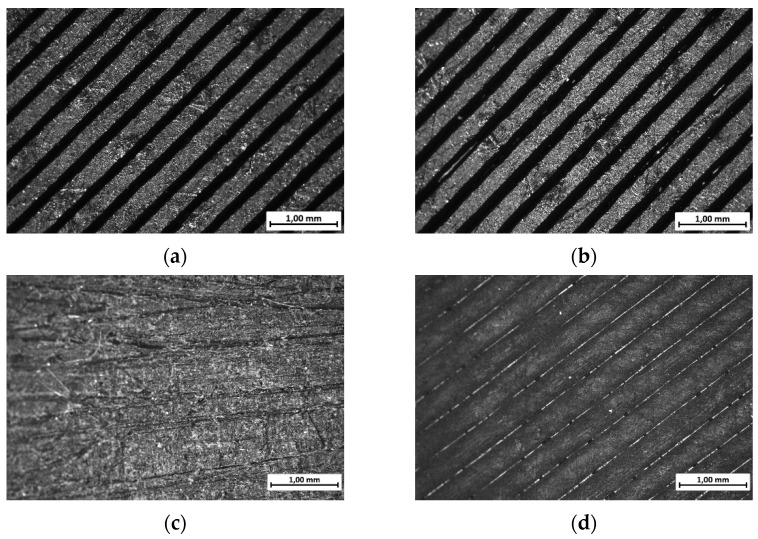
Microscopy analysis of 3D-printed PLA plates’ surface after different surface treatments: (**a**) untreated (UT); (**b**) isopropyl alcohol-cleaned (AL-C); (**c**) mechanical abrasion (MA); (**d**) acetone-cleaned (AC-C).

**Figure 4:**
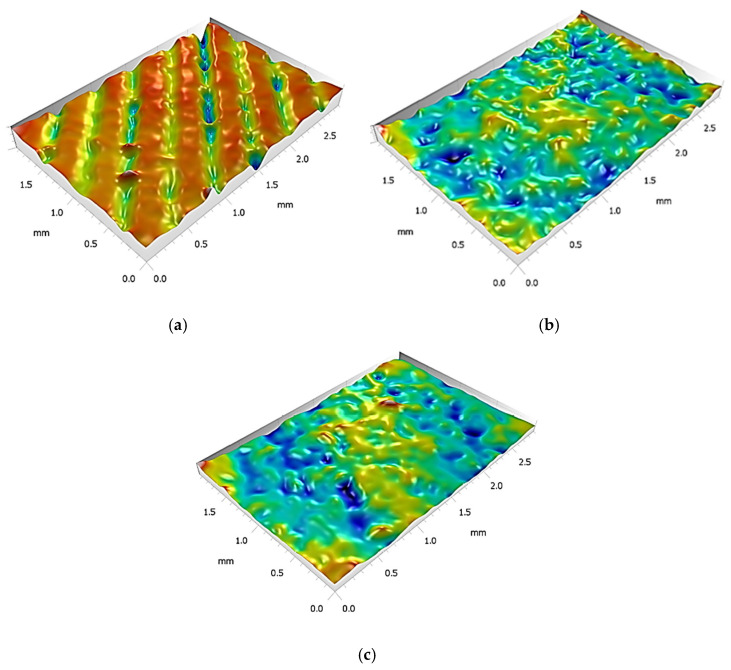
Surface reconstruction obtained via optical image acquisition of PLA substrates subjected to different surface treatments: (**a**) untreated (UT); (**b**) acetone cleaning (AC-C); (**c**) mechanical abrasion (MA). The isopropyl alcohol-treated surface (AL-C) was omitted due to its morphological similarity with the untreated (UT) one.

After the fabrication of the PLA plates via FDM, the specimens were subjected to a detailed morphological characterization using optical microscopy. These analyses were carried out to assess the surface quality and potential defects introduced during the manufacturing process. Subsequently, the plates underwent various post-processing treatments, which were systematically compared in terms of their influence on the surface morphology and structural integrity, enabling an assessment of the effectiveness of each post-treatment approach. The results, summarized in Figure 3, clearly show the influence of the surface preparation. To further quantify these effects, Figure 4 presents the 3D optical surface reconstructions for the untreated (UT), mechanically abraded (MA), and acetone-cleaned (AC-C) samples.

**Figure 5:**
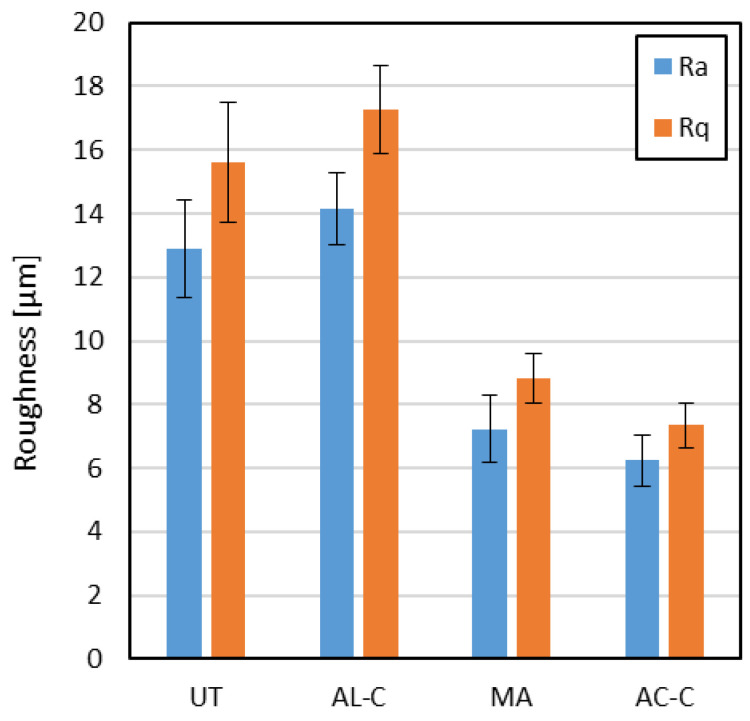
Surface roughness measurements as a function of the surface pre-treatment.

Surface roughness analysis was carried out to evaluate the effectiveness of pre-bonding surface treatments in modifying the morphology of FDM-printed PLA substrates. The results, summarized in Figure 5, clearly show the influence of the surface preparation strategy on both average and root-mean-square roughness parameters.

The untreated surface (UT), corresponding to the top layer of the FDM-printed PLA, exhibited average roughness values of approximately 13 µm (Ra) and 15 µm (Rq). These values are consistent with the inherent layer-by-layer deposition of extruded filaments, where limited resolution and interfacial waviness result in significant surface irregularities.

The surface cleaned with isopropyl alcohol (AL-C) showed a slight increase in both Ra and Rq compared to the untreated condition, reaching values of approximately 14 µm and 17 µm, respectively. This indicates that the solvent did not contribute to surface smoothing; rather, it may have induced selective dissolution or redistribution of low-molecular-weight surface components, potentially exacerbating micro-roughness through re-deposition or differential swelling.

In contrast, the surface subjected to mechanical abrasion followed by compressed-air cleaning (MA) exhibited a marked reduction in roughness, with Ra and Rq values decreasing to approximately 7 µm and 9 µm, respectively. This substantial drop suggests that sanding effectively removed the outermost irregular layer, eliminating high asperities and yielding a more uniform and consistent topography. Although residual texture remains, the distribution of features appears more symmetrical and less variable.

The acetone-cleaned surface (AC-C) showed roughness values comparable to those of the MA-treated surface, with Ra and Rq around 6 µm and 7 µm, respectively. This indicates that acetone, unlike alcohol, had a more significant effect on surface morphology, possibly due to its stronger solvating power toward PLA, which may have caused partial surface reflow or smoothing at the microscale.

Across all conditions, Rq consistently exceeded Ra, confirming the presence of sharp features or localized peaks/valleys that increase the standard deviation of the surface profile. From a bonding perspective, these micrometric discontinuities can improve mechanical interlocking and increase the effective contact area for adhesive bonding.

### 3.2. Adhesion Tests

**Figure 6:**
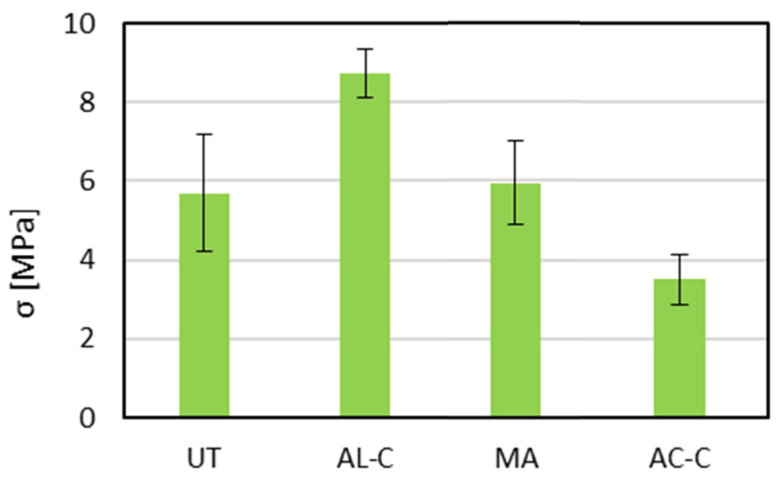
Pull-off strength of adhesive joints as a function of surface treatment.

**Figure 7:**
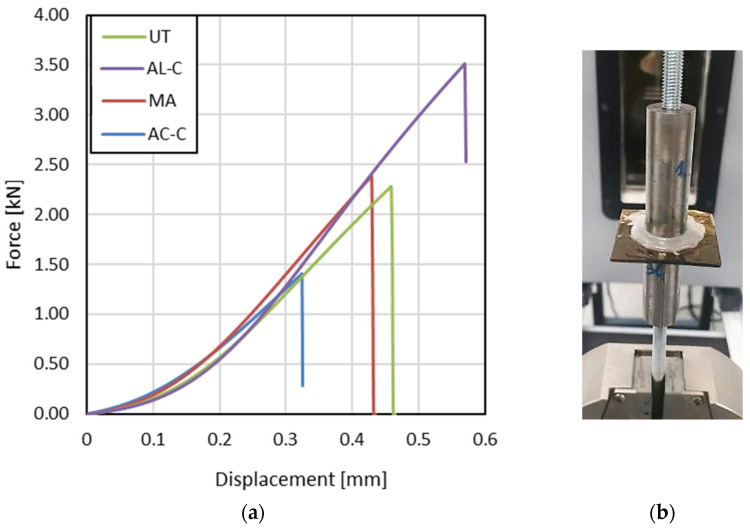
Pull-off test: (**a**) average load–displacement curves; (**b**) particular of a tested specimen.

The pull-off strength results (Figure 6 and Figure 7) highlight the influence of surface preparation on the mechanical performance of PLA-based adhesive joints. The untreated surface (UT) exhibited a moderate average strength of approximately 5.6 MPa, serving as the baseline for comparison. The relatively low performance of this condition underscores the inadequate interfacial interaction between the as-printed PLA surface and the adhesive. Despite the geometrical roughness induced by FDM layering, the lack of chemical activation or cleaning limits the extent of molecular interaction and mechanical interlocking.

The alcohol-cleaned surface (AL-C) demonstrated the highest bond strength, with average pull-off values exceeding 8.5 MPa. This substantial improvement occurred despite a slight increase in surface roughness, as shown in the roughness data, and can thus be primarily attributed to the effective removal of surface contaminants or weak boundary layers. The improved adhesion observed after isopropanol cleaning could be related to enhanced surface wettability, as suggested in previous studies on solvent-cleaned PLA surfaces [15,16,17], although this parameter was not directly measured in the present work.

Conversely, the sample cleaned with acetone (AC-C) showed the lowest performance, with an average strength of approximately 3.5 MPa, even lower than that of the untreated case. This indicates that acetone treatment, while capable of reducing surface roughness, may have adversely altered the surface morphology or chain organization, without evidence of chemical reaction, as confirmed by FTIR analysis. The smoother topography likely contributed to reduced mechanical interlocking, and any adverse chemical effects may have further weakened the interface.

The surface subjected to mechanical abrasion (MA) and compressed-air cleaning yielded intermediate strength values close to 6 MPa. While this condition exhibited reduced surface roughness, the mechanical treatment likely exposed a more uniform polymer layer free from superficial printing defects. However, the absence of chemical cleaning or activation may have limited the enhancement, explaining the lower performance relative to the alcohol-cleaned counterpart. Additionally, the abrasion may have eliminated beneficial morphological features responsible for micro-mechanical interlocking, leading to a less favorable stress distribution at the interface.

In conclusion, the results suggest that isopropyl alcohol cleaning is the most effective method among those evaluated, enhancing adhesion not by altering surface texture but by improving surface cleanliness and potentially modifying interfacial chemistry. In contrast, excessive solvent action or mechanical simplification of the surface can be detrimental to adhesion. These findings underscore the importance of balancing topographical and chemical factors to optimize bonding performance in FDM-printed PLA joints.

## 4. Conclusions

This study investigated the influence of different surface preparation strategies (including mechanical abrasion and solvent treatments) on the adhesive performance of bonded PLA specimens fabricated by FDM. The pull-off test results demonstrated that both mechanical and chemical modifications can significantly enhance the adhesion strength compared to the untreated reference. Among all tested conditions, acetone-treated samples showed the highest pull-off strength, followed by mechanically abraded ones, suggesting the importance of chemical interaction in addition to mechanical interlocking.

The improvement induced by acetone treatment was supported by FTIR analysis, which revealed chemical modifications on the PLA surface, indicating partial hydrolysis and the formation of polar functional groups. This chemical activation is believed to improve wettability and promote stronger interfacial bonding with the epoxy adhesive. On the other hand, mechanical abrasion increased the surface roughness (Ra and Rq values) and led to improved adhesion through enhanced mechanical interlocking, as confirmed by fracture morphology and the observed failure modes.

Although isopropyl-alcohol- and primer-based treatments introduced slight modifications in the pull-off strength, their effects were less pronounced and primarily associated with changes in surface chemistry rather than morphology.

Overall, the findings demonstrate that adhesion strength can be enhanced through tailored surface treatments that promote either morphological or chemical modifications, or both. These insights can inform the development of more effective surface preparation protocols for adhesive bonding in FDM-printed PLA parts, with potential applicability in structural or semi-structural uses. Future studies should investigate the combined effects of mechanical and chemical treatments and evaluate surface free energy through complementary techniques such as contact angle measurements.

## Figures and Tables

**Table 1:** Summary of the printing parameters used for PLA plate fabrication.

Parameter	Value
Nozzle temperature	220 °C
Bed temperature	60 °C
Layer height	0.1 mm
Infill density	100%
Printing speed	60 mm/s
Nozzle diameter	0.4 mm
Raster orientation	±45°

**Table 2:** The experimental plan detailing the surface preparation configurations and associated testing procedures.

Surface Preparation	Label	Number of Specimens
Untreated	UT	5
Isopropyl Alcohol Cleaning	AL-C	5
Mechanical Abrasion	MA	5
Acetone Cleaning	AC-C	5

## Data Availability

The original contributions presented in this study are included in the article. Further inquiries can be directed to the corresponding author.
